# Comparison between the Repression Potency of siRNA Targeting the Coding Region and the 3′-Untranslated Region of mRNA

**DOI:** 10.1155/2013/637850

**Published:** 2013-06-12

**Authors:** Ching-Fang Lai, Chih-Ying Chen, Lo-Chun Au

**Affiliations:** ^1^Institute of Biotechnology in Medicine, Department of Biotechnology and Laboratory Science in Medicine, Yang-Ming University, Taipei 11221, Taiwan; ^2^Department of Medical Research and Education, Taipei Veterans General Hospital, Taipei 11217, Taiwan

## Abstract

Small interfering RNAs (siRNAs) are applied for post-transcriptional gene silencing by binding target mRNA. A target coding region is usually chosen, although the 3′-untranslated region (3′-UTR) can also be a target. This study elucidates whether the coding region or 3′-UTR elicits higher repression. pFLuc and pRLuc are two reporter plasmids. A segment of *FLuc* gene was PCR-amplified and inserted behind the stop codon of the *RLuc* gene of the pRLuc. Similarly, a segment of *RLuc* gene was inserted behind the stop codon of *FLuc*. Two siFLuc and two siRLuc were siRNAs designed to target the central portions of these segments. Therefore, the siRNA encountered the same targets and flanking sequences. Results showed that the two siFLuc elicited higher repression when the *FLuc* segment resided in the coding region. Conversely, the two siRLuc showed higher repression when the *RLuc* segment was in the 3′-UTR. These results indicate that both the coding region and the 3′-UTR can be more effective targets. The thermodynamic stability of the secondary structures was analyzed. The siRNA elicited higher repression in the coding region when the target configuration was stable, and needed to be solved by translation. A siRNA may otherwise favor the target at 3′-UTR.

## 1. Introduction

Two major classes of small regulatory RNAs, small interfering RNAs (siRNAs) and microRNA (miRNAs), are involved in posttranscriptional gene silencing [1]. miRNAs are noncoding endogenous RNAs that direct posttranscriptional repression by binding to partially complementary sites in the 3′-untranslated region (3′-UTR) of the target mRNAs [2]. siRNAs are exogenous RNA designed to bind the target sequence in a perfect match. siRNAs are loaded into the RNA-induced silencing complex (RISC) [3, 4], in which one of the strands is preferentially selected by an Argonaute protein [5], and are guided toward the perfectly paired target. The siRNA/RISC then mediates the endonucleolytic cleavage of the complementary target RNAs and/or causes translational repression [6, 7]. siRNA has become one of the most powerful tools for suppression [8]. However, the efficacy of siRNA varies dramatically [9, 10]. 

Many studies in genomics and sequence analysis approaches, such as in identifying nucleosomes [11], predicting cysteine S-nitrosylation sites [12], identifying recombination spots [13], identifying antimicrobial peptides and their functional types [14], predicting signal propagation during colorectal cancer progression [15], and predicting HIV protease cleavage sites [16], can timely provide very useful information and insights for drug development and hence are widely welcomed by science community. The present study attempted to propose a novel approach for comparing the repression potency of siRNA targeting the coding region and the 3′-untranslated region of mRNA in hopes that the new method can become a useful tool for both basic research and drug development. 

## 2. Materials and Methods

### 2.1. Reporter Vectors Used

pFLuc (pGL3-Control Vector, Promega, Madison, WI, USA), is a firefly luciferase (*FLuc*, coding region 1 nt to 1653 nt) reporter vector. A segment of firefly luciferase gene (91 nt to 454 nt) was PCR-amplified by primers 5′-TATCTAGAGATACGCCCTGGTTCCTG-3′ and 5′-TATCTAGAGATGATAATAATTTTTTGGATG-3′. The introduced *Xba* I restriction cutting sites are underlined. The amplicon was cut with *Xba* I and inserted into the *Xba* I site of the pRLuc (i.e., pRL-TK Vector, Promega) vector. This cutting site is right behind the stop codon of the* Renilla *luciferase gene. The recombinant vector obtained was designated as pRLuc-f. Similarly, pRLuc is a* Renilla *luciferase (*RLuc*, coding region 1 nt to 935 nt) reporter. A segment of *Renilla *luciferase gene (334 nt to 715 nt) was PCR-amplified by primers 5′-ATATCTAGAAGATCATTTTTGTCGGCCA-3′ and 5′-ATATCTAGATTCCTAACAATTTGTACAAC-3′. After cutting by *Xba* I, the amplicon was inserted into the *Xba* I site of the pFLuc vector. This cutting site is right behind the stop codon ofthe firefly luciferase gene. The recombinant vector obtained was designated as pFLuc-r. PSEAP2-Control (Clotech, Inc., Palo Alto, CA, USA), which is a vector encoding secretable alkaline phosphatase (SEAP), was used as an internal control for transfection efficiency. 

### 2.2. siRNA

**Figure 1:**
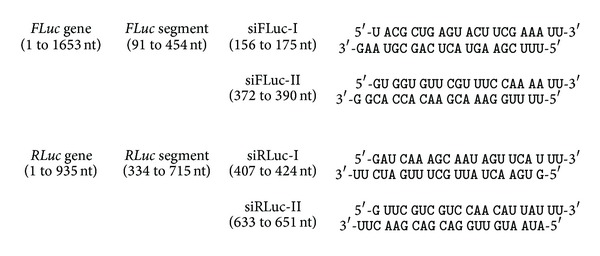
Sequences of siRNA used in this report. The double-stranded sequences of siRNA and their locations in the segments and genes of *FLuc* and *RLuc* are illustrated. The lower strands represent the antisense strands. The numbering starts from nucleotide A of the start codon.

siFLuc and siRLuc are siRNAs targeting the *FLuc* and *RLuc* gene segments described previously. The siFLuc and siRLuc sequences are shown in Figure 1. These siRNAs were obtained from MDBio, Inc. (Taiwan). The siRNAs were purified by HPLC, and their molecular weights were verified by LC/Mass. siRNA negative control (siNC) used as a negative control siRNA was purchased from MDBio, Inc.

### 2.3. Transfection of Reporter Vectors and siRNA

H1299 is a non-small-cell lung carcinoma cell line purchased from the Bioresource Collection and Research Center, Taiwan. The cells grow fast and are easily transfected. H1299 cells were cultured in DMEM containing 10% fetal bovine serum (bought from Gibco BRL, Gaithersburg, MD, USA) in a humidified CO_2_ incubator at 37°C. For transfection, the cells were grown in 6 cm Petri dishes at 70% confluence. One microgram (1 *μ*g) pSEAP2-Control, 1 *μ*g pFLuc (pRLuc, pFLuc-r, or pRLuc-f), and siRNA or siNC (20 nM final concentration) were added to DMEM, bringing it to a final volume of 500 *μ*L. Lipofectamine 2000 (Invitrogen, 10 *μ*L) was added to 490 *μ*L DMEM, and the reagents were well mixed and left undisturbed for 2 min. The two DMEM solutions were mixed and incubated for 20 min. The medium was aspirated from each dish. The mixed DMEM solution and 1 mL DMEM were added to each dish. The cells were incubated in a CO_2_ incubator at 37°C for 4.5 h prior to the addition of complete medium to replace the transfection medium. 

### 2.4. Assays of SEAP, *FLuc*, and *RLuc* Activities

The culture medium was collected 24 h post-transfection and subjected for SEAP assay as described previously [17, 18]. After the withdrawal of the medium, the cells were washed once with 2 mL of 1x PBS and then lysed with 0.5 mL Glo Lysis Buffer (Promega) at room temperature for 5 min, followed by centrifugation to remove the debris. The supernatant was then subjected to *FLuc* and *RLuc* activity assays using Bright-Glo Luciferase Assay System (Promega) and Ready-To-Glow Reporter Assay (Clontech), respectively. Luminescence was quantified by a Tecan Infinite M1000 (Tecan Group Ltd., Switzerland) with the luminescence mode.

### 2.5. Measurement of Fold Repression Caused by siFLuc or siRluc

 Fold repression is defined as B/T, where “B” is the *FLuc* (or *RLuc*) activity divided by its SEAP activity (internal control) for the siNC-treated groups, and “T” is the *FLuc* (or *RLuc*) activity divided by SEAP activity for the siFLuc- (or siRLuc-) treated groups.

## 3. Results and Discussion

**Figure 2:**
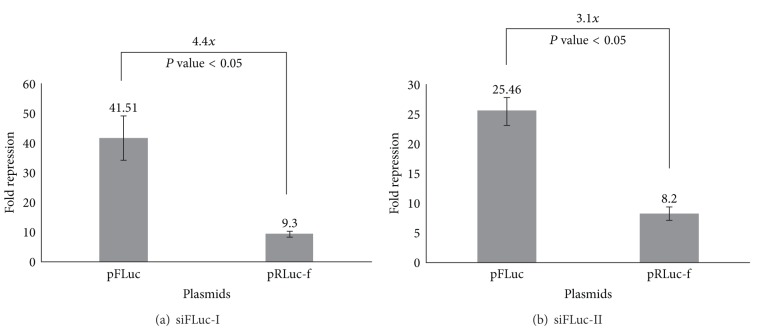
Fold repression caused by (a) siFLuc-I and (b) siFLuc-II on the reporter vectors pFluc and pRLuc-f. For transfection, the H1299 cells were grown in 6 cm Petri dishes at 70% confluence. One microgram (1 *μ*g) pSEAP2-Control, 1 *μ*g pFLuc or pRLuc-f, and siNC or siFLuc (20 nM final concentration) were mixed and transfected into the cells (see Section 2 for details). Measurement of fold repression is mentioned in Section 2. The data presented are the mean ± SD of triplicates. *P* values are also indicated.

**Figure 3:**
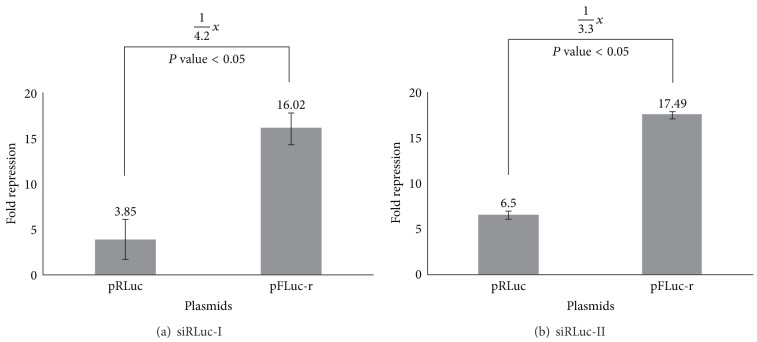
Fold repression caused by (a) siRLuc-I and (b) siRLuc-II on the reporter vectors pRluc and pFLuc-r. For transfection, the H1299 cells were grown in 6 cm Petri dishes at 70% confluence. One microgram (1 *μ*g) pSEAP2-Control, 1 *μ*g pRLuc or pFLuc-r, and siNC or siRLuc (20 nM final concentration) were mixed and transfected into the cells. Measurement of fold repression is mentioned in Section 2. The data presented are the mean ± SD of triplicates. *P* values are also indicated.

**Figure 4:**
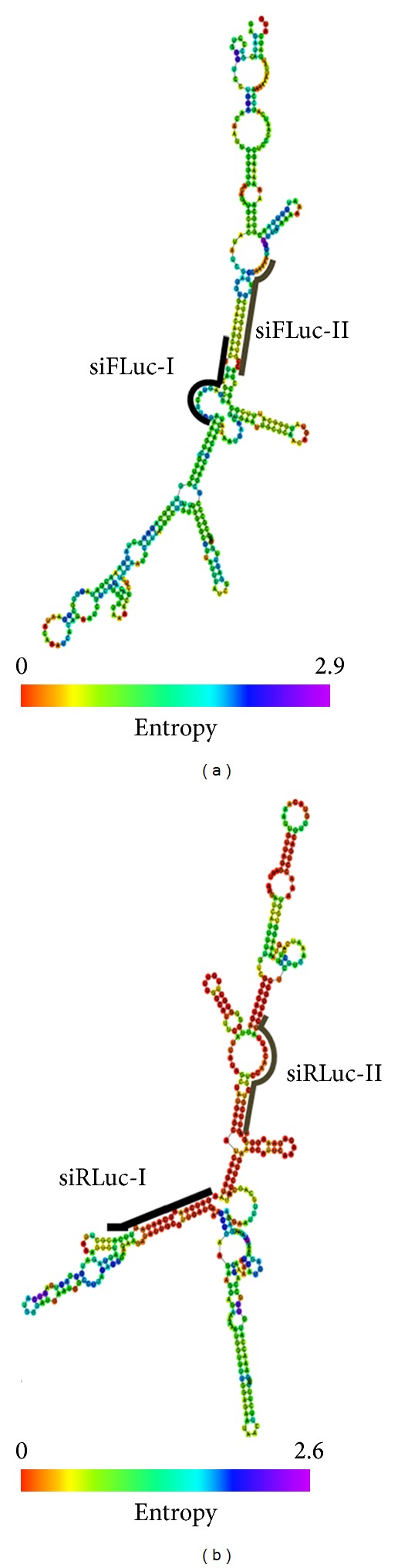
Secondary structures and thermodynamic stabilities of the two mRNA segments. The mRNA segments of the (a) *Fluc* gene (91 nt to 454 nt) and (b) *RLuc* gene (334 nt to 715 nt) were analyzed using an RNAfold program (http://bibiserv.techfak.uni-bielefeld.de/rnafold/). The 2D structures and the entropy assigned to each nucleotide are shown. The siRNA binding sites are indicated by heavy lines.

The cloned segment DNAs in pRLuc-f and pFLuc-r were about 0.4 kb in length. We designed four siRNAs against the middle portion of these segments (see Figure 1). Therefore, the siFLuc binding sites had the same binding and surrounding sequences for both pFLuc and pRLuc-f, except that one resided in the coding region and the other in the 3′-UTR. The same situation was set for siRLuc against the targets on the pRLuc and pFLuc-r vectors. The fold repressions of siFLuc-I on the coding region (pFLuc) and the 3′-UTR (pRLuc-f) are shown in Figure 2(a). The siFLuc-I against the coding region showed significantly higher repression than that against the 3′-UTR. We further evaluated the repression activity of siFLuc-II (Figure 2(b)). siFLuc-II also showed significantly higher repression at the coding region of the *FLuc* segment. These results indicate that the two siFLuc both rendered 3-fold to 4-fold higher repression when targeting the coding region compared when targeting the 3′-UTR. We subsequently tested siRLuc-I and siRLuc-II on their suppression of pRLuc and pFLuc-r (Figures 3(a) and 3(b)). Conversely, the two siRLuc showed higher repression when the *RLuc* segment was in the 3′-UTR. Therefore, both the coding region and the 3′-UTR can be more effective targets, and other factors may affect the outcome. One possible factor is the thermodynamic stabilities of the secondary structures of these two mRNA segments. We analyzed them by using an RNAfold program (http://bibiserv.techfak.uni-bielefeld.de/rnafold/). RNAfold server predicts minimum free energy structures and base pair probabilities from single RNA sequence [18–20]. Fold algorithms and basic options we used are minimum free energy and partition function, with avoiding isolated base pairs. Figures 4(a) and 4(b) show the 2D structures of these two mRNA segments. The entropy assigned to each nucleotide is also shown in these figures. Higher value of positive entropy indicates that these nucleotides are more stably fitted in this structure. Therefore, the 2D structure around the two siFLuc binding sites was more stable than that of the binding site of siRLuc. We speculate that the on-going translational process helped expose the binding sites; therefore, the binding sites at the coding region resulted in higher repression. On the contrary, the configurations of the siRLuc binding sites were thermodynamically less stable and were accessible to siRLuc. The binding sites located in the 3′-UTR, where the interruption by working ribosomes is avoided, rendered higher repression. Most binding sites of endogenous miRNA resided in the 3′-UTR of their target mRNAs [21, 22]. The advantage may be that miRNA-guided translational repression can uncouple the ongoing translation. Consequently, the translational repression of an mRNA can be reflected and fine-tuned by the number of miRNA binding to its 3′-UTR in a cooperative manner [2, 7]. 

Since user-friendly and publicly accessible web servers represent the future direction for developing practically more useful models, simulated methods, or predictors [23], we shall make efforts in our future work to provide a web server for the method presented in this paper.

## 4. Conclusion

In the present report, we illustrate that both the coding region and the 3′-UTR can be highly repressive siRNA targets. The effectiveness of the coding region or the 3′-UTR as a binding site may depend on the thermodynamic stabilities of the secondary structures of the target and the flanking sequence.
